# Binding Modes of Two Scorpion Toxins to the Voltage-Gated Potassium Channel Kv1.3 Revealed from Molecular Dynamics

**DOI:** 10.3390/toxins6072149

**Published:** 2014-07-22

**Authors:** Rong Chen, Shin-Ho Chung

**Affiliations:** Research School of Biology, Australian National University, Canberra, ACT 0200, Australia; E-Mail: shin-ho.chung@anu.edu.au

**Keywords:** margatoxin, hongotoxin, Kv1.3, molecular dynamics, scorpion toxins

## Abstract

Molecular dynamics (MD) simulations are used to examine the binding modes of two scorpion toxins, margatoxin (MgTx) and hongotoxin (HgTx), to the voltage gated K^+^ channel, Kv1.3. Using steered MD simulations, we insert either Lys28 or Lys35 of the toxins into the selectivity filter of the channel. The MgTx-Kv1.3 complex is stable when the side chain of Lys35 from the toxin occludes the channel filter, suggesting that Lys35 is the pore-blocking residue for Kv1.3. In this complex, Lys28 of the toxin forms one additional salt bridge with Asp449 just outside the filter of the channel. On the other hand, HgTx forms a stable complex with Kv1.3 when the side chain of Lys28 but not Lys35 protrudes into the filter of the channel. A survey of all the possible favorable binding modes of HgTx-Kv1.3 is carried out by rotating the toxin at 3° intervals around the channel axis while the position of HgTx-Lys28 relative to the filter is maintained. We identify two possible favorable binding modes: HgTx-Arg24 can interact with either Asp433 or Glu420 on the vestibular wall of the channel. The dissociation constants calculated from the two binding modes of HgTx-Kv1.3 differ by approximately 20 fold, suggesting that the two modes are of similar energetics.

## 1. Introduction

The voltage-gated K^+^ channel Kv1.3, expressed predominantly in human lymphocytes, has been recognized as a promising target for the treatment of certain immune diseases, such as multiple sclerosis, rheumatoid arthritis and type I diabetes [1,2]. When activated by antigens, effector memory T cells express an elevated level of Kv1.3 [3]. By blocking Kv1.3 the activation of T cells can be inhibited and experimentally-induced autoimmune encephalomyelitis in rats can be ameliorated [4].

Various short peptides isolated from venomous animals such as cone snails and scorpions have been identified as potent blockers of Kv1.3 and thus may serve as possible scaffolds for the development of immunosuppressants [5]. These toxins typically consist of 30–40 residues, six of which are cystines constituting three disulfide bonds and the cystine knot motif [6]. A key lysine residue from a toxin physically occludes the selectivity filter of the channel, thereby blocking ion conduction [7]. The toxins are rich in basic residues so that they can form favorable electrostatic interactions with the outer vestibule of Kv1.3, which contains several rings of acidic residues.

Of all the scorpion venom peptides that have been isolated, margatoxin (MgTx) and hongotoxin (HgTx) are among the most potent for Kv1.3. Both toxins inhibit Kv1.3 with picomolar affinities [8,9], but they are equally potent for several other Kv channel isoforms such as Kv1.1 and Kv1.2, which are closely related to Kv1.3 [8,10]. For the pharmacological development of MgTx and HgTx the specificity of the toxins for Kv1.3 needs to be improved. It should be possible to enhance the affinity and selectivity of the toxins once the precise modes of interactions between the toxins and the channel are fully understood. Such detailed interactions involved in the formation of the toxin-channel complexes, once uncovered either experimentally or theoretically, would be valuable for the further development of toxins as drug scaffolds. Numerous experimental and theoretical studies have been carried out to understand the mechanisms of binding and specificity of various toxins for Kv1.3 and other channels [11,12,13,14,15,16,17,18,19,20,21]. However, only a few experimental studies on the structure-activity relationship of MgTx and HgTx have been reported [8,9,10,22]. Accurate models for describing the binding of these toxins to Kv1.3 and other closely-related channels have not yet been established.

Molecular dynamics (MD) simulations and molecular docking methods have been used extensively to examine the binding modes of various toxins to Kv1.3 and other related channels [7,23]. According to these studies and available experimental data [24,25,26], the key lysine residue, typically at position 27 or 28 of the toxin, protrudes into the filter of the homo-tetrameric Kv1.3, forming hydrogen bonds with the carbonyl groups of Tyr447, while another basic residue from the toxin forms a salt bridge with Asp449 or Asp433 on the outer vestibular wall of the channel. The toxin-channel complex is thus stabilized by primarily two strong interactions, one in the filter and the other on the outer vestibule, although in some cases more than one salt bridge may form on the vestibule.

Here, we use MD simulations to examine the binding modes of MgTx and HgTx to Kv1.3. We show that Lys28 of MgTx, which is believed to be the residue physically occluding the filter of Kv1.3, is unable to form stable interactions with the channel pore. MgTx-Lys35, on the other hand, is able to firmly occlude the filter over a substantial period of simulation time, suggesting that MgTx-Lys35 is the pore-blocking residue for Kv1.3. In contrast, HgTx binds firmly to Kv1.3 with its Lys28 side chain protruded into the filter of Kv1.3. A systematic survey of all the possible binding modes of HgTx-Kv1.3 is carried out by rotating the toxin along the channel axis at 3**°** intervals, allowing us to identify two possible binding modes. We show that Arg24 of HgTx is able to form a salt bridge with either Asp449 or Glu420, the two residues which are located in a close proximity on the channel vestibular wall. These simulations demonstrate the power of MD simulations in probing detailed toxin-channel interactions and verifying different models of binding.

## 2. Results and Discussion

### 2.1. Binding of MgTx

We examine the binding of MgTx to Kv1.3 using molecular dynamics with distance restraint. Initially the distance restraint is applied to MgTx-Lys28 and the filter of Kv1.3 because the lysine residue at position 27 or 28 has been found to be the common pore-blocking residue of various scorpion toxins such as kaliotoxin [26] and charybdotoxin [25] for potassium channels. In addition, mutagenesis experiments have shown that MgTx-Lys28 is crucial for the ability of MgTx to inhibit Kv1.3 [10].

**Figure 1:**
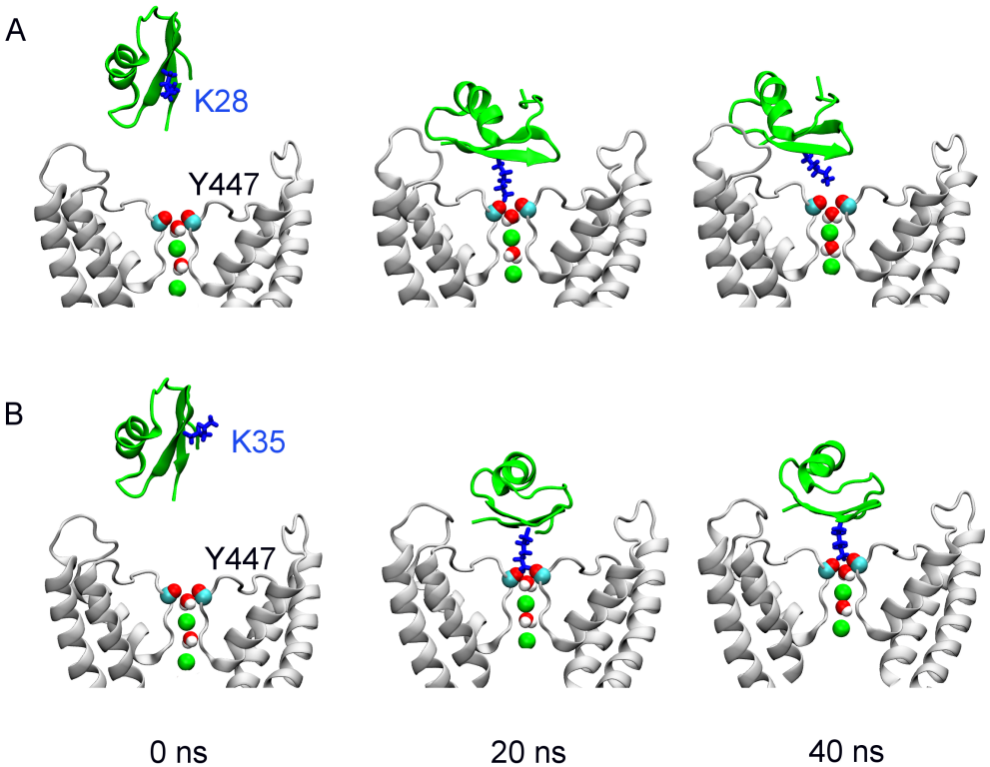
The position of margatoxin (MgTx) (green ribbons) relative to Kv1.3 (white ribbons) at the start (left), and after 20 (middle) and 40 (right) ns of simulation. In (**A**) and (**B**), a distance restraint is applied to draw Lys28 and Lys35 of MgTx, respectively, into the filter of Kv1.3. Two K^+^ ions (green spheres) and two water molecules in the channel filter are highlighted. Only two channel subunits are shown for clarity.

At the start of the simulation, MgTx, the primary structure of which is TIINV-KCTSP-KQCLP-PCKAQ-FGQSA-GAKCM-NGKCK-CYPH, is released in a random orientation in water above the outer vestibule of the channel (Figure 1A, left panel). During the first 20 ns, a distance restraint is applied to the nitrogen atom of the side chain of MgTx-Lys28 and the carbonyl groups of Gly446 in the filter of Kv1.3. The upper boundary of the distance restraint is gradually reduced from 15 to 3 Å, such that the side chain of MgTx-Lys28 is drawn into the filter of the channel. The side chain of MgTx-Lys28 protrudes deeply into the filter of Kv1.3 at 20 ns, forming a hydrogen bond with the carbonyl groups of Kv1.3-Tyr447 (Figure 1A, middle panel). However, once the distance restraint is released, the side chain of MgTx-Lys28 is rejected from the filter rapidly. At 40 ns, the hydrogen bond between MgTx-Lys28 and the filter is completely broken (Figure 1A, right panel). We repeat the simulation a second time with different initial random velocities. In this second simulation the ejection of MgTx-Lys28 from the filter is observed again once the distance restraint is removed (Figure 1B), suggesting that MgTx-Lys28 is not the pore-blocking residue for Kv1.3.

We hypothesize that Lys35, which is in the middle of the β-sheet anti-parallel to the one on which MgTx-Lys28 is located, could be the pore-blocking residue for Kv1.3. To test this conjecture, we repeat the simulations twice, but with the distance restraint now applied to MgTx-Lys35 and Kv1.3-Gly446. In the presence of the distance restraint the chosen residue from MgTx (Lys35) is docked to the channel filter within 20 ns. Afterwards, the distance restraint is released and the simulation is extended to 40 ns. The position of MgTx-Lys35 relative to the filter remains unchanged in both simulations (Figure 1B), consistent with MgTx-Lys35 being the pore-blocking residue for Kv1.3. This MgTx-Kv1.3 complex is stabilized by a number of hydrophobic and electrostatic interactions (Table 1).

**Table 1:** The interaction residue pairs between MgTx and Kv1.3, and between HgTx and Kv1.3 in the bound states of the toxins. The minimum distance (Å) of each residue pair is given. Standard deviations are also shown. The umbrella windows corresponding to the wells in the potential of mean force (PMF) profiles in Figure 2B and Figure 5 are used for analysis. Note in the complex of MgTx-Kv1.3, Lys33 of the toxin forms a hydrogen bond with the backbone carbonyl group of Asp449 of the channel.

MgTx-Kv1.3	Average distance	HgTx-Kv1.3 (R24-D433)	Average distance	HgTx-Kv1.3 (R24-E420)	Average distance
I2-H451	2.3 ± 0.3	I23-T425	2.6 ± 0.3	P10-H451	2.7 ± 0.6
K28-D449	1.7 ± 0.1	R24-D433	1.7 ± 0.1	R24-E420	1.8 ± 0.3
M30-M450	2.3 ± 0.2	K28-Y447	1.8 ± 0.2	K28-Y447	1.8 ± 0.2
K33-D449	2.0 ± 0.4	M30-H451	2.5 ± 0.3	M30-H451	2.4 ± 0.3
K35-Y447	1.8 ± 0.1	K35-D449	1.8 ± 0.3	K35-D449	1.8 ± 0.4
Y37-F428	2.8 ± 0.4	Y37-H451	2.5 ± 0.3	Y37-H451	2.4 ± 0.3
P38-V453	3.3 ± 0.7	H39-V453	2.4 ± 0.3	H39-M450	2.5 ± 0.3

**Figure 2:**
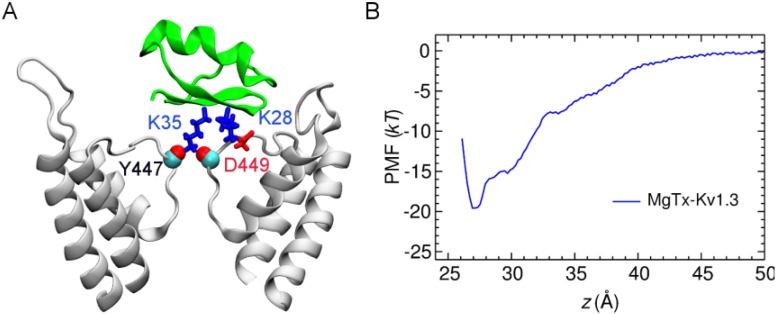
(**A**) MgTx (green ribbons) bound to the outer vestibule of Kv1.3 (white ribbons) at 40 ns; (**B**) PMF profile along the channel axis (*z*) derived from the binding mode in (**A**).

The complex structure of MgTx-Kv1.3 with MgTx-Lys35 occluding the filter reveals that MgTx-Lys28 forms a salt bridge with Kv1.3-Asp449 just outside the filter (Figure 2A). The affinity of MgTx would be compromised if Lys28 were mutated to a neutral residue and the Lys28-Asp449 salt bridge broken. This is consistent with the low ability of K28A mutant MgTx to block Kv1.3 observed experimentally [10]. We construct the potential of mean force (PMF) profile of the binding of the MgTx-Kv1.3 complex predicted from our simulations. The PMF profile shows a well depth of 19.5 kT (Figure 2B), corresponding to a *K*_d_ (dissociation constant) of 20 nM, which is within two orders of magnitude to the experimental values of 80–100 pM [9,10]. Thus, the simulations are consistent with experiment and suggest that both Lys28 and Lys35 of MgTx are crucial for Kv1.3 inhibition, because MgTx-Lys28 forms a salt bridge with Kv1.3-Asp449 and MgTx-Lys35 physically occludes the filter.

### 2.2. Binding of HgTx

HgTx (primary structure TVIDV-KCTSP-KQCLP-PCKAQ-FGIRA-GAKCM-NGKCK-CYPH) inhibits Kv1.3 potently with a picomolar affinity, and is highly selective for Kv1.3 over Kv1.5, which is expressed in the heart [8]. The Lys28 residue of HgTx is important for its function [22], similar to various other scorpion toxins. Based on the available experimental evidence, a distance restraint is applied between HgTx-Lys28 and Gly446 from the filter of Kv1.3 during the first 20 ns of simulation to predict the binding mode of HgTx-Kv1.3. Again at the start of the simulation the toxin is placed in water 15 Å above the channel. The simulation is repeated a second time with different random initial velocities.

**Figure 3:**
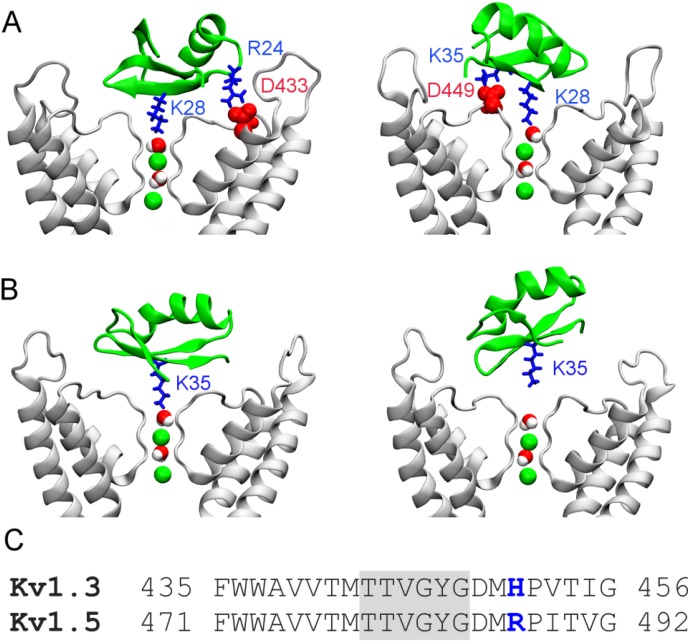
HgTx (green ribbons) bound to Kv1.3 (white ribbons) predicted from molecular dynamics with distance restraint. Two water molecules and two K^+^ ions (green spheres) in the channel filter are shown. In (**A**) and (**B**), the snapshots are taken from the simulations in which HgTx-Lys28 and HgTx-Lys35 are chosen as the pore-blocking residue, respectively; In (**A**), the salt bridges Arg24-Asp433 (left) and Lys35-Asp449 (right) of the HgTx-Kv1.3 complex at 40 ns are highlighted; In (**B**), the position of HgTx relative to Kv1.3 at 20 (left) and 40 (right) ns is shown; In (**C**), a sequence alignment of Kv1.3 and Kv1.5 near the filter region (shadowed) is given.

The two simulations consistently show that HgTx binds firmly to Kv1.3 with the HgTx-Lys28 side chain protruded deeply into the channel filter at 40 ns, as shown in Figure 3A. The side chain of HgTx-Lys28 without the distance restraint remains in the filter until the simulation is terminated at 40 ns. The salt bridge between HgTx-Arg24 and Kv1.3-Asp433, which is common to various closely related toxin-channel complexes [7], stabilizes the binding (Figure 3A). However, this salt bridge is not present in the MgTx-Kv1.3 complex (Figure 2), as MgTx does not carry an arginine. Instead, the Lys28-Asp449 salt bridge is present in MgTx-Kv1.3. In addition to Arg24-Asp433, a second salt bridge is observed between HgTx-Lys35 and Kv1.3-Asp449 (Figure 3A). A number of hydrophobic interactions are also observed (Table 1). Similar to MgTx, the key residues of HgTx involved in binding Kv1.3 are also primarily from the two β-strands at the *C*-terminus (residues 24–39), consistent with other scorpion toxins selective for Kv1.3 [27].

To verify if HgTx-Lys28 is the pore-blocking residue for Kv1.3, we repeat the simulation twice with the distance restraints applied to HgTx-Lys35. In the presense of the distance restraint, the side chain of HgTx-Lys35 is drawn deeply into the filter during the first 20 ns (Figure 3B, left panel), forming a hydrogen bond with the carbonyl groups of Kv1.3-Tyr447. No additional hydrogen bond or salt bridges are observed, indicating that the binding is weak. Thus, once the distance restraint is removed, the side chain of HgTx-Lys35 is rapidly rejected from the filter (Figure 3B, right panel), suggesting that HgTx-Lys35 is unlikely to be the pore-blocking residue for Kv1.3.

In our model of HgTx-Kv1.3, HgTx-Lys35 forms a salt bridge with Kv1.3-Asp449 and is in close proximity to Kv1.3-His451. This histidine residue at position 451 in Kv1.3 is replaced by a charged arginine in Kv1.5 (Figure 3C). Thus, the HgTx-Kv1.5 complex would not be as strong as HgTx-Kv1.3, because of the unfavorable interactions between HgTx-Lys35 and Kv1.5-Arg487 if HgTx binds to the two channels in the same orientation. This prediction is consistent with the high selectivity of HgTx for Kv1.3 over Kv1.5 observed experimentally [8]. The presence of Kv1.5-Arg487 also suggests that the binding affinity can be improved by introducing acidic residues into the toxin. Scorpion toxins rich in acidic residues have been shown to be active on the Kv7.1 channel that is also highly resistant to basic toxins like HgTx and MgTx [28]. Recently, it has been shown that arginine residues in the P-loop turret region of Ca^2+^-activated K^+^ channels (K_Ca_) contribute to the selectivity of scorpion toxins [29]. Thus, it appears that electroatstic interactions are crucial for toxin binding and specificity [12,30], and the presence of several basic residues, either located in the P-loop turret or near the selectivity filter, is important for the resistance of certain subtypes of K^+^ channels such as Kv1.5 and K_Ca_2.3 to scorpion toxins that are highly basic.

### 2.3. Two Binding Modes of HgTx-Kv1.3

In previous computational studies of scorpion toxins and Kv1.3, a general assumption is that a predominant binding mode exists between the toxin and the channel. However, different models for the same toxin-channel system have been reported [17,31]. It is possible that for certain toxins multiple binding modes of similar energetics exist and as such it is important to consider all these binding modes in describing toxin action. Here, using HgTx-Kv1.3 as a model system, we carry out a systematic survey on the binding modes of HgTx-Kv1.3, based on the assumption that HgTx-Lys28 is the pore-blocking residue for Kv1.3.

**Figure 4:**
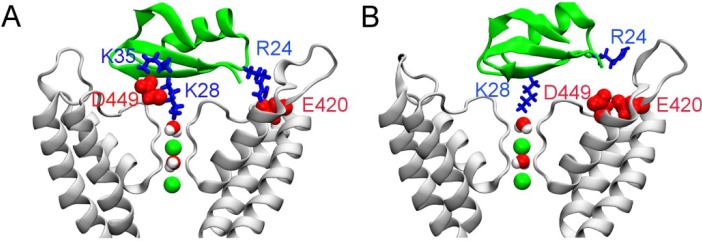
Position of HgTx (green ribbons) relative to the Kv1.3 (white ribbons) predicted from molecular dynamics. Two water molecules and two K^+^ ions (green spheres) in the channel filter are shown. In (**A**) and (**B**), the orientation of HgTx is rotated by 30° and 60° along the channel axis from the binding mode of Figure 3A.

From the bound position of HgTx-Kv1.3 predicted from biased MD, we rotate HgTx at 3° intervals along the channel axis while maintaining the position of HgTx-Lys28 relative to the filter. Since the channel has fourfold symmetry, the rotation only spans 90° and a total of 30 structures are generated. Each structure is then relaxed for 10 ns using MD, with the orientation of the toxin along the channel axis harmonically restraint. The structures of HgTx-Kv1.3 in which the toxin is rotated by 30° and 60° along the channel axis are shown in Figure 4. With a 30° rotation, the Lys35-Asp449 salt bridge is retained, but HgTx-Arg24 now forms a salt bridge with Kv1.3-Glu420 rather than Kv1.3-Asp433 (Figure 4A). The most important toxin-channel interacting residue pairs in this binding mode are given in Table 1. On the other hand, both the Lys35-Asp449 and Arg24-Asp433 salt bridges are broken with a 60° rotation (Figure 4B).

**Figure 5:**
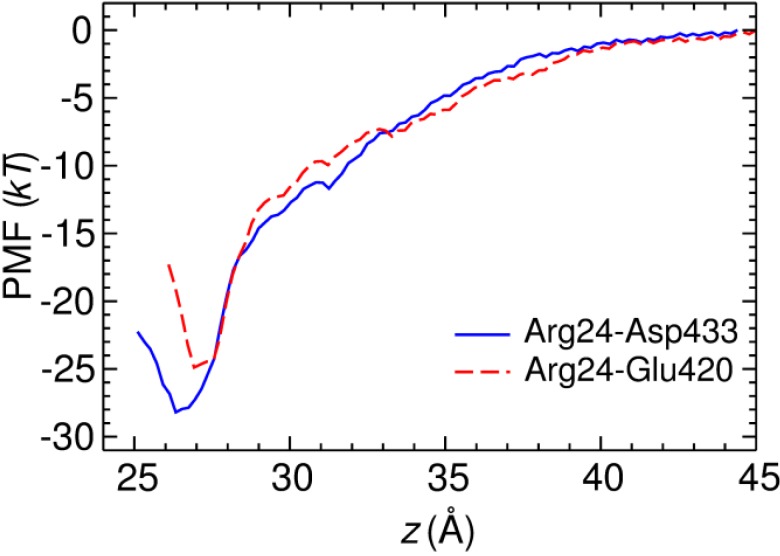
PMF profiles for the two binding modes of HgTx-Kv1.3 in which the salt bridges Arg24-Asp433 and Arg24-Glu420 are formed, respectively. The reaction coordinate is the center of mass (COM) distance between the backbones of the toxin and the channel along the channel axis (*z*).

The simulations of HgTx-Kv1.3 suggest two favorable binding modes, in which HgTx-Arg24 forms a salt bridge with Kv1.3-Glu420 and Kv1.3-Asp433, respectively. To ascertain which mode is more favorable, we deduce the potential of mean force (PMF) profile of toxin binding. Since the configurational space sampled over a limited period of simulation time is highly restrained by the initial binding mode, the PMF profile obtained reflects the binding energy of this particular binding mode and can thus be used to ascertain the relative strength of different modes. The binding mode of Figure 4B is not considered because no salt bridge is present indicating unfavorable toxin-channel interactions. The two PMF profiles deduced from the binding modes of Figure 3 and Figure 4A virtually overlap when the toxin is not fully bound to the channel (*z* > 28 Å, Figure 5). This indicates that the PMF profiles are well converged, because the same PMF is predicted from different initial conditions when the toxin is not fully bound. The depth of the two PMF profiles differs by 3.4 kT, with the profile of the Arg24-Asp433 mode being slightly deeper. The *K*_d_ values calculated from the PMF profiles are 8 and 180 pM for the Arg24-Asp433 and Arg24-Glu420 modes, respectively. Both values are within 10-fold of the experimental value of 86 pM [8], suggesting that two binding modes of similar energetics may exist for HgTx-Kv1.3.

## 3. Experimental Section

### 3.1. Molecular Dynamics Simulations

The equilibrated structure of Kv1.3 embedded in a 1-palmitoyl-2-oleoyl-*sn*-glycero-3-phosphocholine bilayer and a box of explicit water is taken from our previous study [31]. Two water molecules and two K^+^ ions are placed in the selectivity filter, corresponding to that observed in the crystal structure of charybdotoxin bound to a K^+^ channel [24]. The solution structures 1MTX [32] and 1HLY [22] are used for MgTx and HgTx, respectively. MD simulations with distance restraint, which have been applied successfully to closely-related systems [33], are applied to predict the binding modes of each toxin to Kv1.3. At the start of the simulation the toxin is released in a random orientation in water 15 Å above the position when it is fully bound to the channel. Each simulation is repeated a second time to ascertain if the binding mode predicted is reproducible.

All MD simulations are performed under periodic boundary conditions using NAMD 2.9 [34]. The CHARMM36 force fields for lipids and proteins and the TIP3P model for water are used [35,36]. The switch and cutoff distances for short-range interactions are set to 8.0 Å and 12.0 Å, respectively. The long-range electrostatic interactions are accounted for using the particle mesh Ewald method, with a maximum grid spacing of 1.0 Å. Bond lengths are constrained allowing a time step of 2 fs to be used. The temperature and pressure are maintained constant at 300 K and 1 atm, respectively. The pressure coupling is semiisotropic. Trajectories are saved every 20 ps for analysis.

### 3.2. Potential of Mean Force Calculations

PMF calculations are performed using umbrella sampling. The starting structures of the umbrella windows spaced at 0.5 Å intervals are generated using steered molecular dynamics. The reaction coordinate is the center of mass (COM) distance between the backbone of the toxin and the backbone of the channel along the channel axis. The biasing potential of each window is 30 kcal/mol/Å^2^. A flat-bottom harmonic restraint is applied to maintain the COM of the toxin within a cylinder of 8 Å in radius centered on the central axis of the channel. The restraining potential is always zero when the toxin is bound to the channel. Each window is simulated for up to 13 ns until the depth of the PMF profile changes by less than 0.5 kT over the last 1 ns. The *K*_d_ value is derived from the PMF profile according to the equation derived elsewhere [7,37].

## 4. Conclusions

MD simulations are used to examine the binding of two scorpion venom peptides, MgTx and HgTx, to the voltage-gated K^+^ channel Kv1.3, which is a target for immunosuppression. The simulations suggest that MgTx-Lys35 occludes the ion conduction pathway of the channel on the formation of the MgTx-Kv1.3 complex, while Lys28 of the toxin stabilizes the complex by forming an additional salt bridge with Asp449, located just outside of the selectivity filter. We note here that Lys28 of MgTx does not form a salt bridge with Asp433 of Kv1.3. In contrast, HgTx-Lys28 is the key residue that blocks the filter of Kv1.3. Once the side chain of HgTx-Lys28 protrudes into the filter of Kv1.3, the complex is stabilized by either the Arg24-Asp433 or the Arg24-Glu420 salt bridge. The dissociation constants of the two binding modes of HgTx-Kv1.3 estimated from PMF calculations differ by ~20 fold, suggesting that the energetics of the two modes do not differ dramatically. This finding is similar to the previous calculations on charybdotoxin and K_Ca_3.1, for which two binding modes of similar PMFs were identified [38]. Thus, multiple binding modes may be required to describe the interactions of certain toxin-channel systems such as HgTx-Kv1.3.

Experimental techniques such as mutagenesis have been widely used to study the functional surface of various toxins and their receptor sites on channels. Structures of several toxin-channel complexes have been deduced from X-ray crystallography and NMR [24,25,26,39,40], shedding lights on the mechanisms of toxin action. On the other hand, the details of molecular interactions that these experiments can probe are limited. For example, only ensemble averages of given properties can be obtained, and the interacting residue pairs between a toxin and a channel are not directly observable experimentally, but inferred from the results obtained by various experimental manipulations. In the case of mutagenesis, several assumptions are made implicitly in data interpretation. It is often assumed that the toxin has a unique predominant binding mode, and a simple mutation does not alter the orientation of toxin binding or the overall structure of the toxin. However, both experimental and theoretical studies have suggested that these assumptions are invalid in certain systems. For example, a single mutation can induce significant structural changes to a toxin [41,42,43] and multiple binding modes of similar energetics are required to describe the action of certain toxins [44,45,46]. Therefore, experimental data can be difficult to interpret. In the present work, we show that MD simulations allow atomic models of toxin-channel complexes to be constructed using available experimental data as restraints. These models would provide structural basis for the interpretation of available experimental data and can be verified by new experiments.
